# A publicly accessible database for *Clostridioides difficile* genome sequences supports tracing of transmission chains and epidemics

**DOI:** 10.1099/mgen.0.000410

**Published:** 2020-07-29

**Authors:** Martinique Frentrup, Zhemin Zhou, Matthias Steglich, Jan P. Meier-Kolthoff, Markus Göker, Thomas Riedel, Boyke Bunk, Cathrin Spröer, Jörg Overmann, Marion Blaschitz, Alexander Indra, Lutz von Müller, Thomas A. Kohl, Stefan Niemann, Christian Seyboldt, Frank Klawonn, Nitin Kumar, Trevor D. Lawley, Sergio García-Fernández, Rafael Cantón, Rosa del Campo, Ortrud Zimmermann, Uwe Groß, Mark Achtman, Ulrich Nübel

**Affiliations:** ^1^​ Leibniz Institute DSMZ, Braunschweig, Germany; ^2^​ Warwick Medical School, University of Warwick, UK; ^3^​ German Center for Infection Research (DZIF), Partner site Hannover-Braunschweig, Germany; ^4^​ Braunschweig Integrated Center of Systems Biology (BRICS), Technical University, Braunschweig, Germany; ^5^​ AGES-Austrian Agency for Health and Food Safety, Vienna, Austria; ^6^​ Christophorus-Kliniken, Coesfeld, Germany; ^7^​ Research Center Borstel, Germany; ^8^​ German Center for Infection Research (DZIF), Partner site Hamburg-Lübeck-Borstel, Germany; ^9^​ Friedrich-Loeffler-Institut, Jena, Germany; ^10^​ Biostatistics, Helmholtz Centre for Infection Research, Braunschweig, Germany; ^11^​ Institute for Information Engineering, Ostfalia University, Wolfenbüttel, Germany; ^12^​ Wellcome Sanger Institute, Hinxton, UK; ^13^​ Servicio de Microbiología, Hospital Universitario Ramón y Cajal, and Instituto Ramón y Cajal de Investigación Sanitaria (IRYCIS), Madrid, Spain; ^14^​ Red Española de Investigación en Patología Infecciosa (REIPI), Madrid, Spain; ^15^​ University Medical Center Göttingen, Germany

**Keywords:** *Clostridioides (Clostridium) difficile*, nosocomial infection, genomic population structure, outbreak, cgMLST, hierarchical clustering

## Abstract

*
Clostridioides difficile* is the primary infectious cause of antibiotic-associated diarrhea. Local transmissions and international outbreaks of this pathogen have been previously elucidated by bacterial whole-genome sequencing, but comparative genomic analyses at the global scale were hampered by the lack of specific bioinformatic tools. Here we introduce a publicly accessible database within EnteroBase (http://enterobase.warwick.ac.uk) that automatically retrieves and assembles *C. difficile* short-reads from the public domain, and calls alleles for core-genome multilocus sequence typing (cgMLST). We demonstrate that comparable levels of resolution and precision are attained by EnteroBase cgMLST and single-nucleotide polymorphism analysis. EnteroBase currently contains 18 254 quality-controlled *C. difficile* genomes, which have been assigned to hierarchical sets of single-linkage clusters by cgMLST distances. This hierarchical clustering is used to identify and name populations of *C. difficile* at all epidemiological levels, from recent transmission chains through to epidemic and endemic strains. Moreover, it puts newly collected isolates into phylogenetic and epidemiological context by identifying related strains among all previously published genome data. For example, HC2 clusters (i.e. chains of genomes with pairwise distances of up to two cgMLST alleles) were statistically associated with specific hospitals (*P*<10^−4^) or single wards (*P*=0.01) within hospitals, indicating they represented local transmission clusters. We also detected several HC2 clusters spanning more than one hospital that by retrospective epidemiological analysis were confirmed to be associated with inter-hospital patient transfers. In contrast, clustering at level HC150 correlated with *k*-mer-based classification and was largely compatible with PCR ribotyping, thus enabling comparisons to earlier surveillance data. EnteroBase enables contextual interpretation of a growing collection of assembled, quality-controlled *C. difficile* genome sequences and their associated metadata. Hierarchical clustering rapidly identifies database entries that are related at multiple levels of genetic distance, facilitating communication among researchers, clinicians and public-health officials who are combatting disease caused by *C. difficile *.

## Data Summary

All genome sequencing data were submitted to the European Nucleotide Archive (www.ebi.ac.uk/ena) under study numbers PRJEB33768, PRJEB33779 and PRJEB33780. The *Clostridioides* database within EnteroBase is publicly accessible at http://enterobase.warwick.ac.uk. In addition, stand-alone versions of all EnteroBase tools are available at https://github.com/zheminzhou/EToKi.

**Impact Statement**

*
Clostridioides difficile* is a major cause of healthcare-associated diarrhea and causes large infection outbreaks. Whole-genome sequencing is increasingly applied for genotyping *C. difficile *, with the objectives to monitor and curb the pathogen's spread. We present a publicly accessible database for quality-controlled genome sequences from *C. difficile* that enables contextual interpretation of newly collected isolates by identifying related strains among published data. It also provides a nomenclature for genomic types to facilitate communication about transmission chains, epidemics and phylogenetic lineages. Finally, we demonstrate that genome-based hierarchical clustering is largely compatible with previously used molecular typing techniques, thus enabling comparisons to earlier surveillance data.

## Introduction

The anaerobic gut bacterium *Clostridioides difficile* (formerly *Clostridium difficile *) [1] is the primary cause of antibiotic-associated diarrhea in Europe and North America [2]. Molecular genotyping of *C. difficile* isolates has demonstrated international dissemination of diverse strains through healthcare systems [3–5], the community [6] and livestock production facilities [7, 8]. Previously, genotyping was commonly performed by PCR ribotyping or DNA macrorestriction. More recent publications have documented that genome-wide single-nucleotide polymorphisms (SNPs) from whole-genome sequences provide improved discrimination, and such analyses have enabled dramatic progress in our understanding of the emergence and spread of epidemic strains [9–12] and the epidemiology of local transmission [13, 14]. Eyre and colleagues have argued that transmission of *C. difficile* isolates within a hospital environment can be recognized with high probability as chains of genomes, which differ by up to two SNPs whereas genomes, which differ by at least ten genomic SNPs represent unrelated bacteria [13, 15]. However, SNP analyses require sophisticated bioinformatic tools and are difficult to standardize [16, 17]. A convenient alternative to SNP-based genotyping is offered by the commercial software SeqSphere, which implements a core-genome multilocus sequence typing scheme (cgMLST) for the analysis of genomic diversity in *C. difficile* [18] and other organisms. Indeed, cgMLST [18] confirmed the prior conclusion from genomic SNP analyses [19] that a common clone of *C. difficile* had been isolated over two successive years at a hospital in China [18]. However, a recent quantitative comparison of the two methods showed that SeqSphere's cgMLST achieved a low predictive value (41 %) for identifying isolate pairs that were closely related by the ≤2 SNPs' criterion [20]. cgMLST of genomic sequences of a variety of bacterial pathogens can also be performed with EnteroBase (http://enterobase.warwick.ac.uk/), which has been developed over the last few years with the goal of facilitating genomic analyses by microbiologists [21]. EnteroBase automatically retrieves Illumina short-read sequences from public short-read archives. It uses a consistent assembly pipeline to automatically assemble these short-reads into draft genomes consisting of multiple contigs, and presents the assembled genomes together with their metadata for public access [22]. It also performs the same procedures on sequencing data uploaded by its registered users. Assembled genomes that pass quality control are genotyped by MLST at the levels of seven-gene MLST, ribosomal MLST (rMLST), cgMLST and whole-genome MLST (wgMLST) [21, 22]. EnteroBase supports subsequent analyses based on either SNPs or cgMLST alleles using the GrapeTree or Dendrogram visualization tools [23]. EnteroBase also assigns these genotypes to populations by hierarchical clustering (HierCC), which supports the identification of close relatives at the global level [22]. Originally, EnteroBase was restricted to the bacterial genera *Salmonella, Escherichia, Yersinia* and *Moraxella* but since January 2018, EnteroBase has included a database for genomes and their metadata for the genus *Clostridioides *. In June 2020, EnteroBase contained 18 254 draft genomes of *C. difficile* plus one genome of *C. mangenotii *. These included over 900 unpublished draft genomes that were sequenced at the Leibniz Institute DSMZ, as well as 80 complete genome sequences based on Pacific Biosciences plus Illumina sequencing technologies. It also included 862 unpublished draft genomes that were sequenced at the Wellcome Sanger Institute.

Here we show that comparable levels of resolution and precision are attained by EnteroBase cgMLST as by SNP analyses. We also summarize the genomic diversity that accumulated during recurring infections within single patients as well as transmission chains within individual hospitals and between neighbouring hospitals in Germany, and show that it can be detected by HierCC. We also demonstrate that HierCC can be used to identify bacterial populations at various epidemiological levels ranging from recent transmission chains through to epidemic and endemic spread, and relate these HierCC clusters to genotypes that were identified by PCR ribotyping and *k*-mer-based diversity analysis. These observations indicate that cgMLST and HierCC within EnteroBase can provide a common language for communications and interactions by the global community who is combatting disease caused by *C. difficile *.

## Results

### Implementation of MLST schemes in EnteroBase

**Fig. 1.**
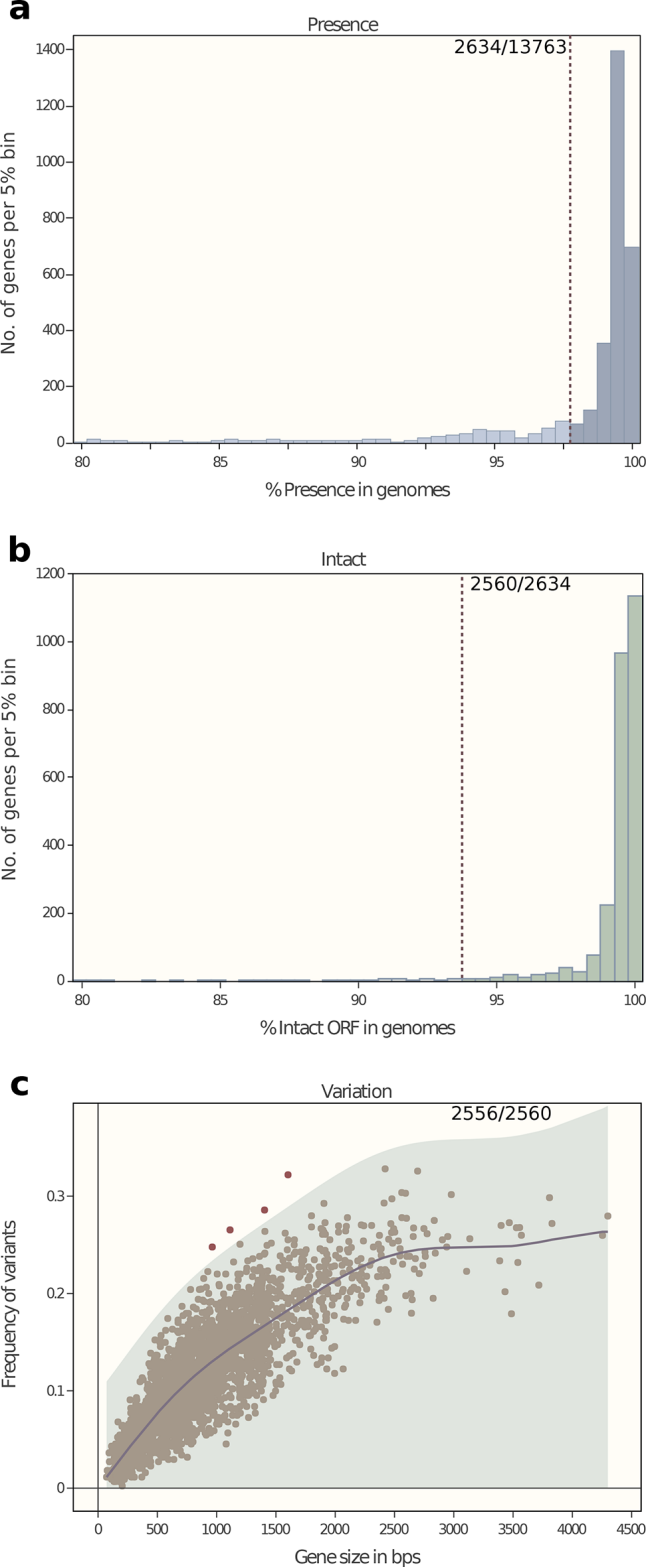
Criteria for inclusion in a cgMLST scheme of a subset of wgMLST genes based on their properties in a reference set of 442 genomes (https://tinyurl.com/Cdiff-ref). (a) Numbers of genes versus frequency (% presence) within the reference set. In total, 2634 genes satisfied the cut-off criterion of ≥98 % presence (dashed line). (b) Numbers of genes versus intact ORF (% intact ORF) within the 2634 genes from (a). Overall, 2560 genes satisfied the cut-off criterion of ≥94 % intact ORF (dashed line). (c) Frequency of allelic variants versus gene size among the 2560 genes from (b). The genetic diversity was calculated using the GaussianProcessRegressor function in the sklearn module in Python. This function calculates the Gaussian process regression of the frequency of genetic variants on gene sizes, using a linear combination of a radial basis function kernel (RBF) and a white kernel [57]. The shadowed region shows a single-tailed 99.9% confidence interval (≤3 sigma) of the prediction. Altogether, 2556 loci fell within this area and were retained for the cgMLST scheme, while four were excluded due to excessive numbers of alleles.

cgMLST in EnteroBase consists of a defined subset of genes within a whole-genome MLST scheme that represents all single-copy orthologues within the pan-genome of a representative set of bacterial isolates. To this end, we assembled the draft genomes of 5232 isolates of *C. difficile* from public short-read archives, and assigned them to ribosomal sequence types (rSTs) according to rMLST, which indexes diversity at 53 loci encoding ribosomal protein subunits on the basis of existing exemplar alleles at PubMLST [24]. We then created a reference set of 442 genomes consisting of one genome of *C. mangenotii* [1], 18 complete genomes from GenBank, 81 closed genomes from our work and the draft genome with the smallest number of contigs from each of the 343 rSTs (https://tinyurl.com/Cdiff-ref). The *Clostridioides* pan-genome was calculated with PEPPA [25] and used to define a wgMLST scheme consisting of 13 763 genetic loci (http://enterobase.warwick.ac.uk/species/clostridium/download_data). EnteroBase uses the wgMLST scheme to call loci and alleles from each assembly, and extracts the allelic assignments for the subsets corresponding to cgMLST, rMLST and seven-gene MLST from those allelic calls [22]. The cgMLST subset consists of 2556 core genes, which were present in ≥98% of the reference set, intact in ≥94% and were not excessively divergent (Fig. 1).

### Comparison of cgMLST and SNPs for analyses of transmission chains

We compared the numbers of cgMLST allelic differences and the numbers of non-recombinant SNPs in isolates from multiple epidemiological chains. These included 176 isolates from four patients with recurring CDI (* C. difficile* infection), 63 isolates from four transmission chains in multiple hospitals [14, 19, 26], and a comprehensive sample of 1158 isolates collected over several years in four hospitals in Oxfordshire, UK [13]. A strong linear relationship (R^2^, 0.71–0.93) was found in all three analyses between the pairwise differences in cgMLST alleles and non-recombinant SNPs (Fig. S1, available in the online version of this article). The slope of the regression lines was close to 1.0, indicating a 1 : 1 increase in cgMLST allelic differences with numbers of SNPs. The same data were also investigated with cgMLST calculated with the commercial program SeqSphere [18], with similar correlation coefficients but a lower slope due to lesser discriminatory power of the SeqSphere cgMLST scheme (lower panels in Fig. S1).

**Fig. 2.**
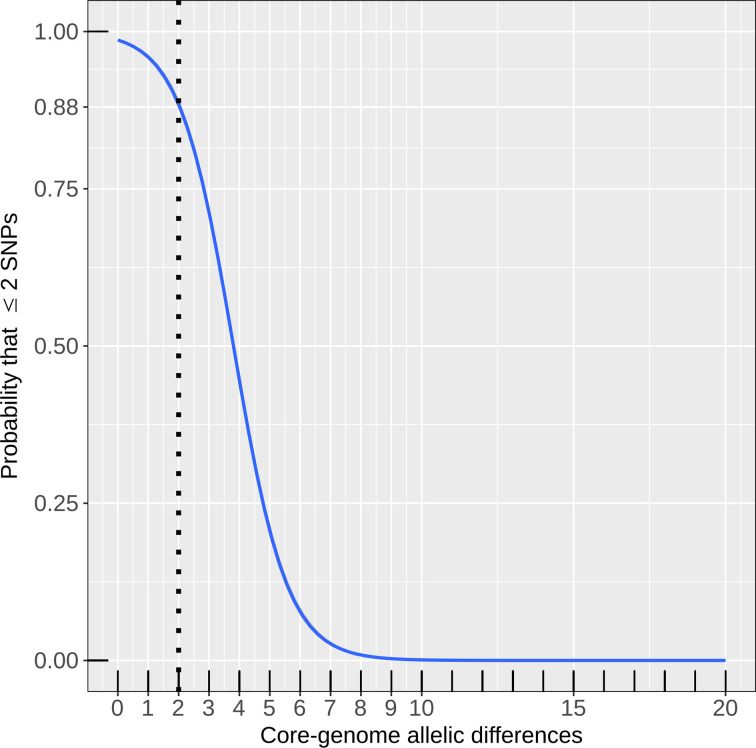
Binary logistic regression model to determine the probability that two genomes are related at ≤2 SNPs, given a certain difference in their cgMLST allelic profiles, based on the Oxfordshire dataset [13]. The number of SNPs was encoded as a binary dependent variable (1 if ≤2 SNPs, 0 if otherwise) and the number of allelic differences was used as a predictor variable.

Eyre *et al.* [13] concluded that direct transmission between two hospital patients can be detected because their bacterial genomes differ by two SNPs or less. Our analysis indicated that these transmission chains in the Oxfordshire dataset would also have been recognized by cgMLST in EnteroBase. Genomes that differed by two cgMLST alleles usually also differed by ≤2 SNPs according to a binary logistic regression model (probability=89%; 95% confidence interval, 88–89%) (Fig. 2). Of 3807 pairs of genomes with ≤2 allelic differences, 3474 also differed by ≤2 SNPs, yielding a positive predictive value of 91 % for identifying isolate pairs with ≤2 SNPs by EnteroBase cgMLST and a sensitivity of 62 % (≤2 cgMLST allelic differences were found in 3474 of 5707 pairs with ≤2 SNPs). The comparable values for SeqSphere were 78 % positive predictive value and 99% sensitivity.

We also compared the genetic distances between 242 genomes from Oxfordshire, which had been isolated during the initial 6 months and 916 genomes from the actual testing period (April 2008 to March 2011) [13]. Overall, 35% (318/916) of the latter genomes matched at least one genome collected earlier by two or less EnteroBase cgMLST alleles and 34% (316/916) matched an earlier genome by ≤2 SNPs. The two sets of genomes were 89% concordant. Thus, cgMLST is equivalent to SNP analysis for detecting inter-patient transmission chains.

### Hierarchical clustering for tracing local and regional spread

SNP analyses are computer intensive, and are only feasible with limited numbers of genomes [27]. cgMLST-based relationships can be analysed for up to 100 000 genomes with GrapeTree, but analyses involving more than 10 000 genomes remain computer intensive [23]. EnteroBase implements single-linkage hierarchical clustering (HierCC V1) of cgMLST data in pairwise comparisons at multiple levels of relationship after excluding missing data [22]. These are designated as HC0 for hierarchical clusters of indistinguishable core-genome sequence types (cgSTs), HC2 for clusters with pairwise distances of up to two cgMLST alleles, etc*.* EnteroBase presents cluster assignments for *C. difficile* at the levels of HC0, HC2, HC5, HC10, HC20, HC50, HC100, HC150, HC200, HC500, HC950, HC200 and HC2500. Here we address the nature of the genetic relationships that are associated with these multiple levels of HierCC among 13 515 publicly available *C. difficile* genomes, and examine which levels of pairwise allelic distances correspond to epidemic outbreaks and to endemic populations.

**Fig. 3.**
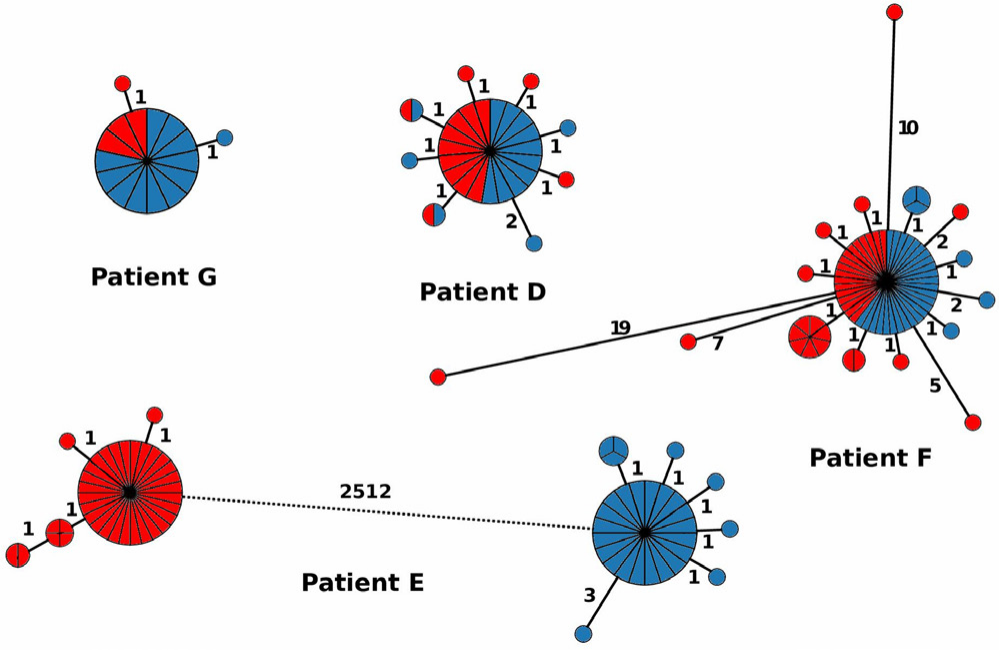
Minimum-spanning trees indicating the population structure of *C. difficile* in four patients with recurrent CDI episodes. Red, first episode; blue, second episode.

In our analyses of 176 *C. difficile* isolates from four patients with two recurrent episodes of CDI, multiple genomes were assigned to patient-specific HC2 clusters, some of which were isolated from the initial episode as well as the recurrence 80–153 days later (Fig. 3, patients D, F and G; 4 to 36 isolates had been collected per episode; Table S1). For these patients, relapsing disease likely reflected continued colonization after initially successful therapy. However, some isolates from patient F differed by 12–21 cgMLST alleles from the bulk population (Fig. 3), which indicates that the patient was co-infected simultaneously with multiple related strains. In patient E, the two genomes from the two CDI episodes differed by >2000 allelic differences (Fig. 3), which indicates that the second incident of CDI represented an independent infection with an unrelated strain. Hence, discrimination between relapse and reinfection based on cgMLST appears to be straightforward except that two episodes of CDI might arise by reinfection with identical strains from an environment that is heavily contaminated with *C. difficile* spores [28]. We note that the time intervals (16–22 weeks) investigated here exceeded the currently recommended threshold of 8 weeks for surveillance-based detection of CDI relapses [29, 30] but still yielded almost identical strains in three of four patients.

**Fig. 4.**
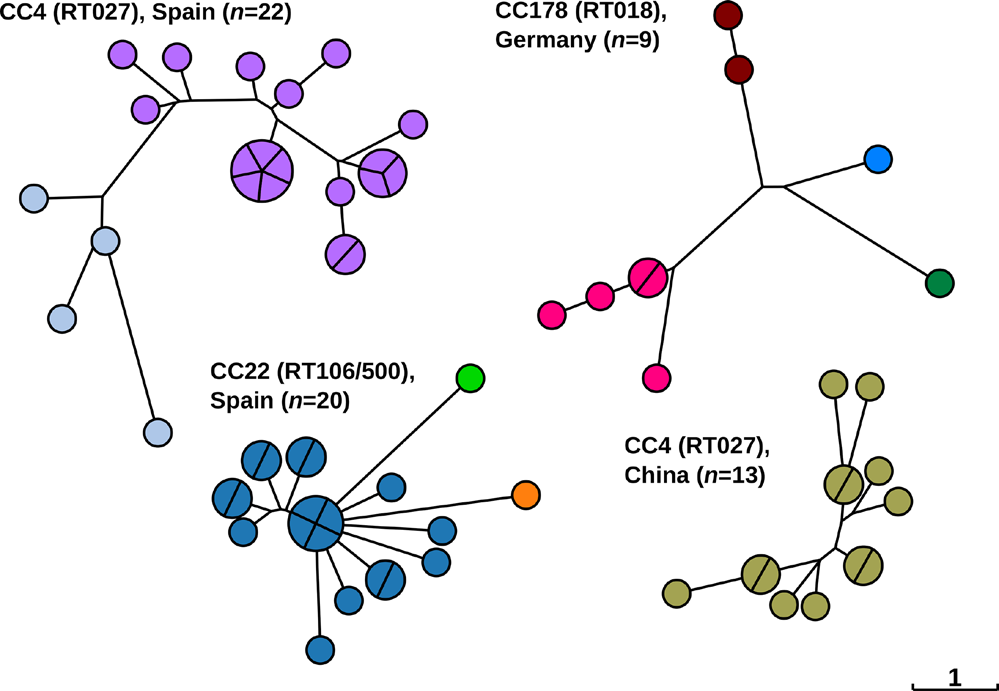
Neighbour-joining trees based on cgMLST showing the phylogenetic relationships among *C. difficile* isolates from previously published CDI outbreaks as indicated [14, 19, 26]. Nodes are coloured by HC2. CC, cgST complex, i.e. related at level HC150; RT, PCR ribotype. The scale, indicating one allelic difference, applies to all trees.

Our examinations of multiple local outbreaks have revealed individual, outbreak-specific HC2 clusters. However it is also conceivable that multiple HC2 clusters might be isolated from a single epidemiological outbreak due to the accumulation of genetic diversity over time. Alternatively, multiple HC2 clusters within a single outbreak may represent the absence of crucial links due to incomplete sampling. Incomplete sampling of outbreaks is not unlikely because asymptomatic patients are only rarely examined for colonization with *C. difficile* [31–33] even though they may constitute an important reservoir for transmission. Indeed, some of the outbreaks investigated here did consist of more than one HC2 cluster (Fig. 4). For example, nine isolates from a recently reported ribotype 018 (RT018) outbreak in Germany [26] encompassed four related HC2 clusters, and outbreaks with RT027 and RT106 in a hospital in Spain [14] were each affiliated with two or three HC2 clusters (Fig. 4).

**Fig. 5.**
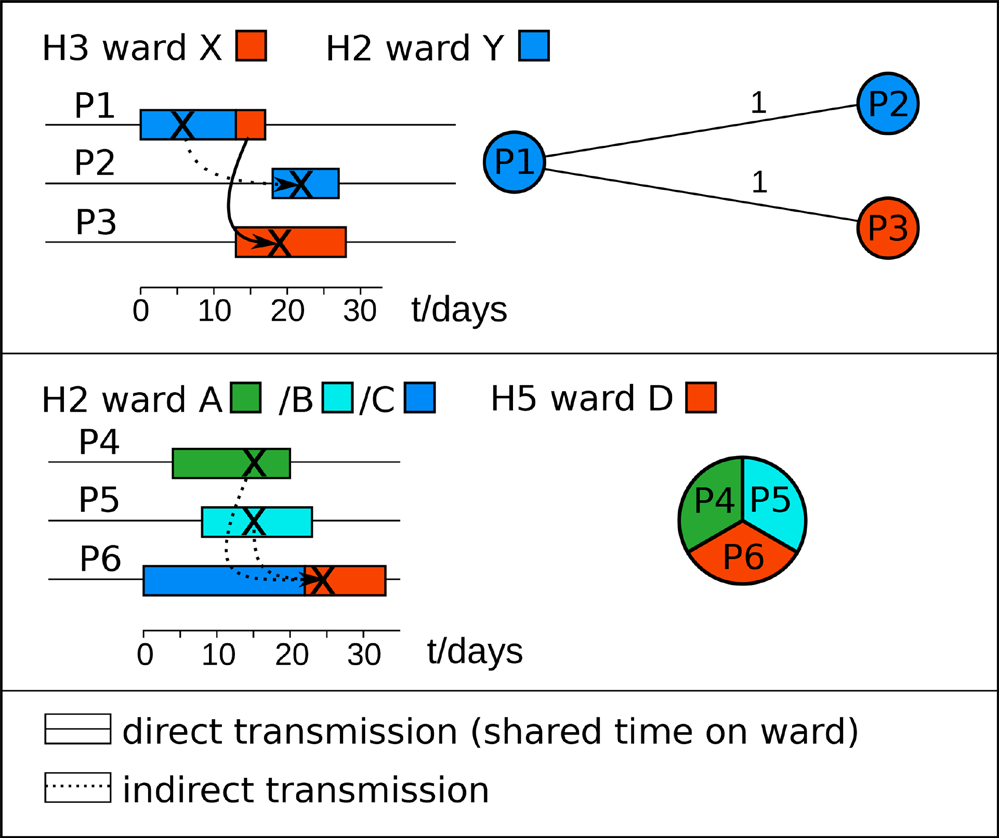
Timelines of two transmission chains, discovered retrospectively through inspection of files from CDI patients with closely related *C. difficile* isolates (HC2). Colours indicate hospital wards, 'X' indicate diagnosis of CDI, and arrows indicate presumed transmission pathways. Minimum-spanning trees indicating genomic distances among *C. difficile* isolates are shown on the right. Upper panel: patient P1 was diagnosed with CDI in hospital H2 and transferred to hospital H3 15 days later. Another five and 6 days later, respectively, patients P2 in hospital H2 and P3 in hospital H3 got diagnosed with CDI with closely related strains. Both these patients were on the same wards as the initial patient, who probably had been the source for the pathogen. Since there was no temporal overlap between patient P2 and the other patients in hospital H2, transmission may have occurred indirectly, possibly through environmental contamination. Lower panel: another putative transmission chain involved three patients that had shared time in hospital H2. Patients P4 and P5 got diagnosed with CDI on the same day after they had shared 7 days in this hospital, albeit on different medical wards. The third patient developed CDI with the same *C. difficile* cgST 4 days after being transferred to another hospital (h5), but had previously stayed at hospital H2 during the time when CDI got diagnosed in the first two patients. Since the three patients stayed on different wards in hospital H2, transmission presumably occurred indirectly.

We identified 23 HC2 clusters encompassing 133 genome sequences in a dataset of 309 *C. difficile* genome sequences collected from CDI patients in six neighbouring hospitals in Germany. These HC2 clusters were associated with individual hospitals (Χ^2^, *P*=8.6×10^−5^; Shannon entropy, *P*=4.2×10^−5^) and even with single wards in these hospitals (Χ^2^, *P*=0.01; Shannon entropy, *P*=6.2×10^−3^). We investigated whether these HC2 clusters reflected the local spread of *C. difficile* within institutions by retrospective analyses of patient location data. Sixty six patients (50 %) were found to have had ward contacts with another patient with the same HC2 cluster (median time interval between ward occupancy: 63 days; range, 0 to 521). These results are consistent with the direct transmission on wards of *C. difficile* isolates of the same HC2 cluster (Fig. 5). For patients such as P1 and P2 where the shared ward contacts were separated in time (Fig. 5), transmission may have occurred indirectly through asymptomatically colonized patients or from a common reservoir, such as environmental spore contamination [14, 31, 32]. We also detected 15 HC2 clusters that included isolates from two or more hospitals in the region. Subsequent analyses of patient location data confirmed that some of these HC2 clusters were associated with patient transferrals between the hospitals (Fig. 5). Hence, hierarchical clustering of *C. difficile* genome sequences in conjunction with retrospective analysis of patient movements revealed multiple likely nosocomial transmission events, none of which had been detected previously by routine surveillance.

### Hierarchical clustering for identification of epidemic strains and endemic populations

**Fig. 6.**
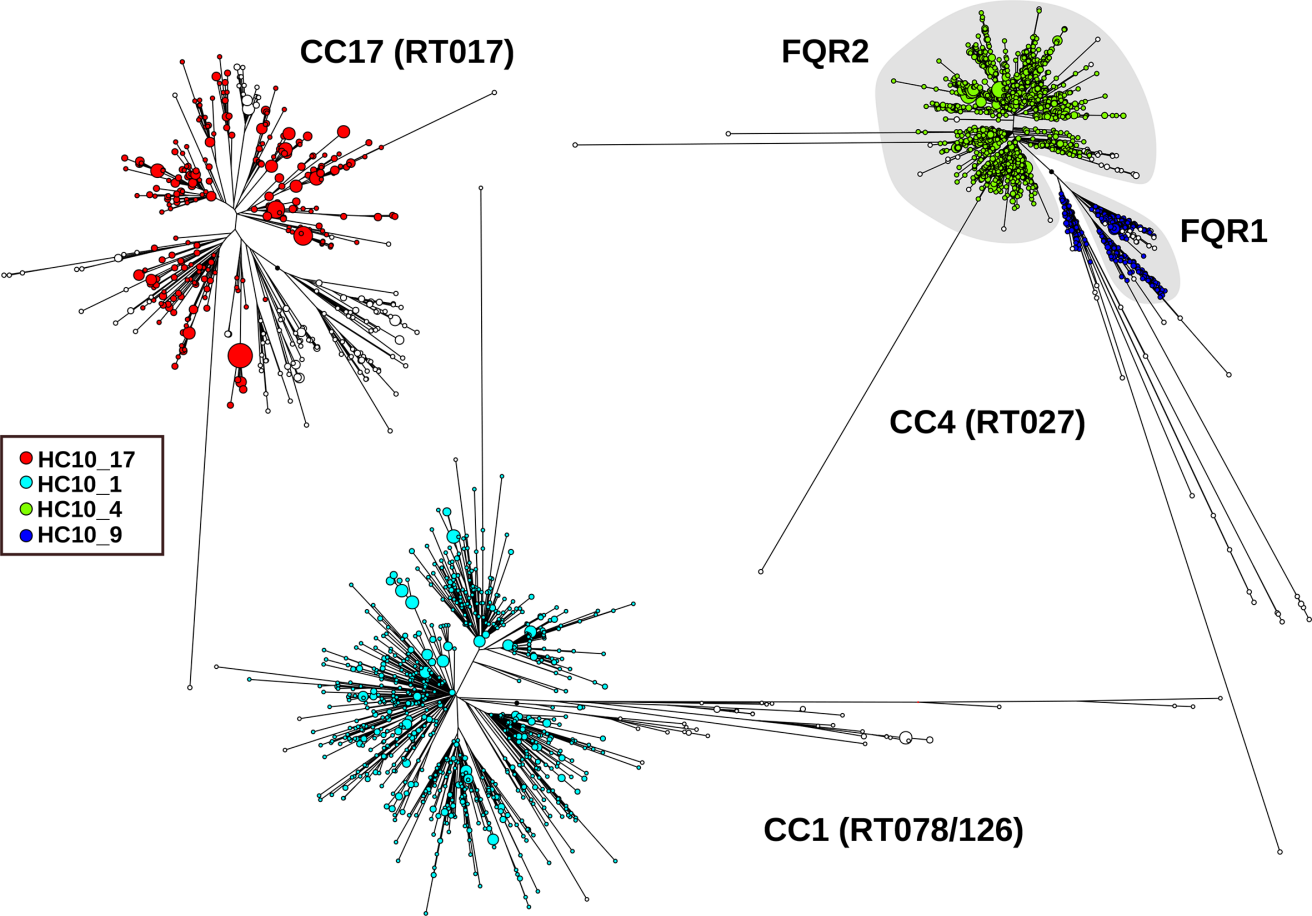
Phylogenetic structure of three international *C. difficile* epidemics, each of which has spread for about 25 years [9, 11]. Within each epidemic, the majority of isolates is related at level HC10, as indicated by the colours. CC, cgST complex, i.e. related at level HC150; RT, PCR ribotype.

International epidemic spread of *C. difficile* over up to 25 years has been inferred previously on the basis of molecular epidemiology with lower resolution techniques [34]. For multiple representatives of those epidemic strains in EnteroBase, the majority of these epidemic groups corresponded to HC10 clusters, including epidemic RT017 [11] (HC10_17), the two fluoroquinolone-resistant lineages of RT027 [9] (HC10_4, HC10_9), or livestock-associated RT078/126 [35] (HC10_1) (Fig. 6).

Endemic populations have also been described by ribotyping and phylogenetic analyses, some of which have acted as sources for the emergence of epidemic strains [2, 9]. Many endemic populations seem to be represented by HC150 clusters. Clustering at HC150 was well supported statistically (Fig. S2), and the frequency distribution of pairwise genomic distances indicated that multiple database entries clustered at <150 cgMLST allelic differences (Fig. S3). HC150 clusters also correlated well with *k*-mer-based classification [36]. When applied to the dataset of 309 *C. difficile* genomes from six hospitals in Germany, the two methods implemented in EnteroBase and PopPUNK found 51 and 48 clusters, respectively, the majority of which coincided (adjusted Rand coefficient, 0.97).

**Fig. 7.**
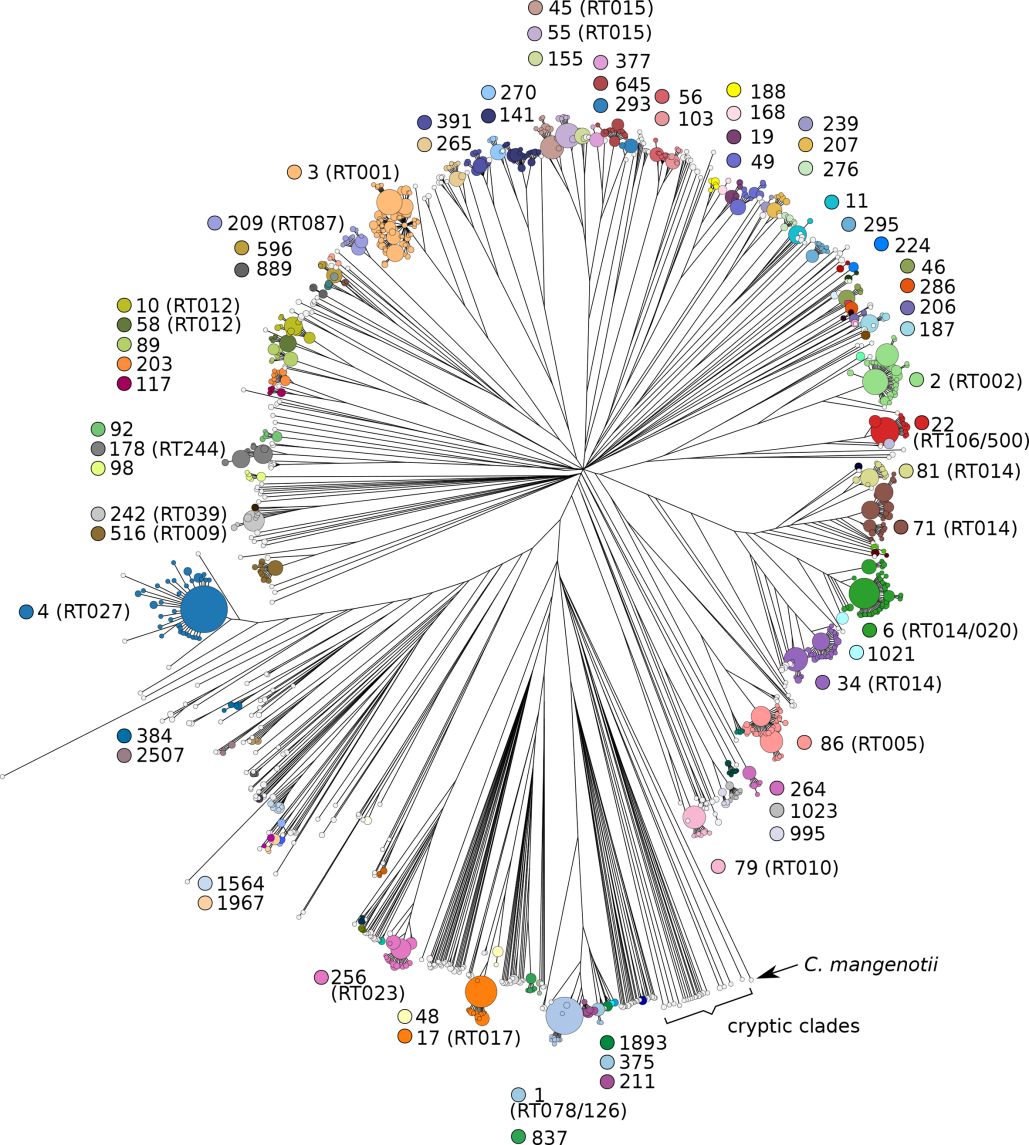
Rapid-neighbour-joining phylogenetic tree based on cgMLST variation from 13 515 *C. difficile* genomes. Colours and numerals indicate CCs (HC150 clusters) with ≥10 entries, and information on predominant PCR ribotypes is provided in brackets.

A cgMLST-based phylogenetic tree of 13515 *C. difficile* genomes showed 201 well-separated HC150 clusters, each encompassing a set of related isolates, plus 209 singletons (Fig. 7). Because these HC150 clusters are based on cgMLST genetic distances, we refer to them as 'cgST complexes', abbreviated as CCs. Genomes from each of the major CCs have been collected over many years in multiple countries, indicating their long-term persistence over wide geographic ranges (Table 1).

**Table 1.** Characteristics of cgST complexes (CC) with ≥100 entries

CC (HC150)	PCR Ribotype	Number of entries	Sampling years	Number of countries	% isolates in HC2>2^1^	% isolates from animal hosts
4	027	2669	1985–2018	27	77	0
1	078, 126, 066	1222	1994–2018	26	61	17
17	017	769	1990–2017	24	64	0
3	001	768	1980–2017	16	62	0
6	020, 404	768	1995–2017	14	43	1
2	002	702	2006–2017	15	51	1
22	106, 500	531	1997–2017	7	59	3
86	005	468	1980–2017	8	41	0
34	014	421	1995–2017	10	35	0
55	015	318	2006–2017	6	37	0
71	014, 020	315	2004–2017	16	40	1
145	015	284	2006–2016	7	39	0
256	023	268	2001–2015	6	40	0
79	010	249	2003–2018	7	53	3
178	018, 356	243	2006–2017	7	52	0
242	039	199	2008–2017	4	58	1
10	012	159	1996–2017	7	52	0
88	014	132	1996–2016	9	33	8
11	070	110	2006–2017	6	32	0
187	054	109	2007–2018	6	47	0
141	001, 026	107	2007–2016	2	7	0
391	081	105	1996–2016	4	31	0
49	011, 056, 446	103	2001–2017	5	35	0

^1^isolates in HC2 clusters with >2 entries.

**Fig. 8.**
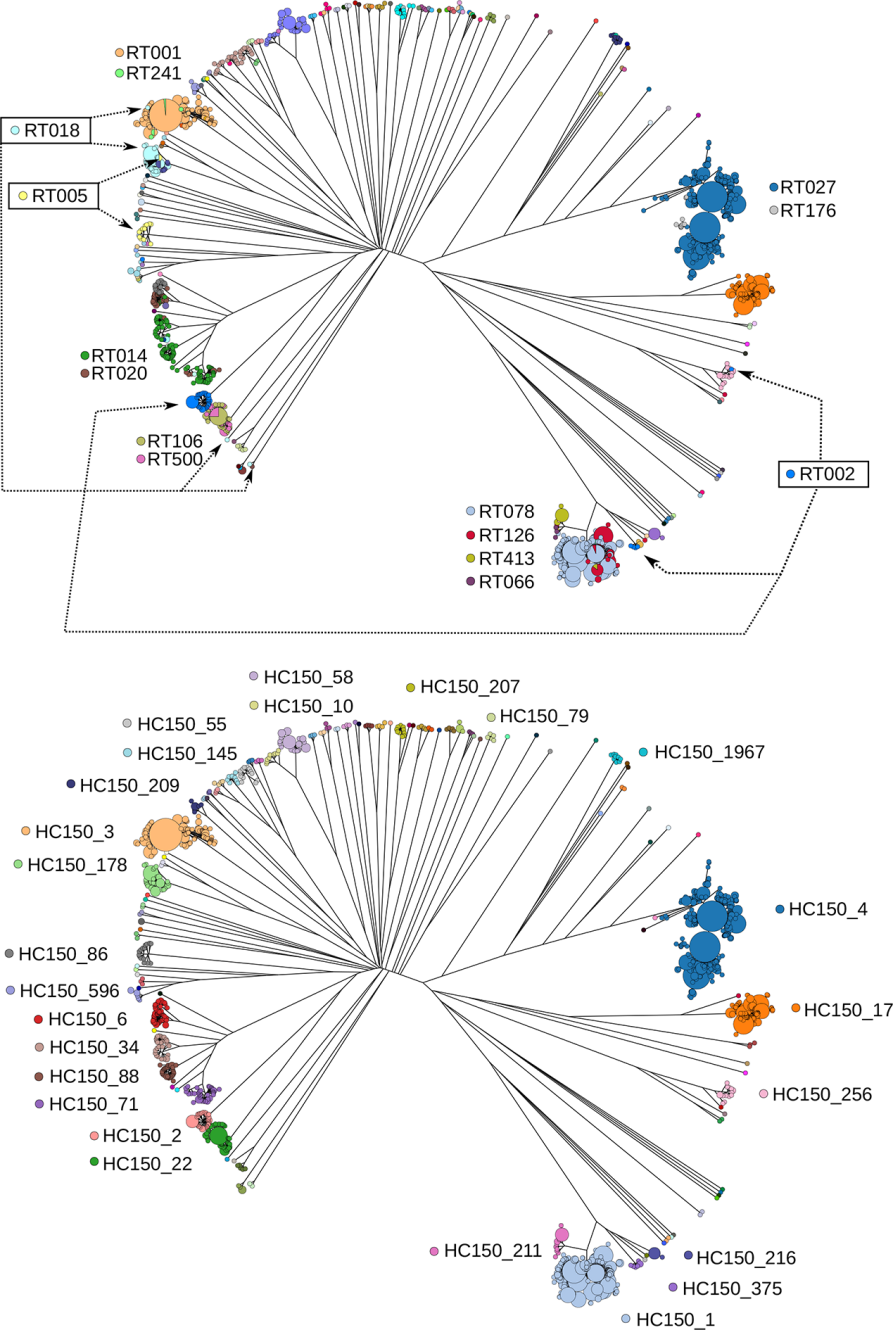
Rapid-neighbour-joining phylogenetic tree based on cgMLST variation from 2263 *C. difficile* genomes, for which PCR ribotyping information is available. Upper panel: nodes are coloured by PCR ribotype as indicated. Lower panel: nodes are coloured by CC (HC150 clusters).

We compared HC150 clustering with PCR ribotyping for 2263 genomes spanning 84 PCR ribotypes for which PCR ribotyping data were available in EnteroBase. These included 905 genomes, which we ribotyped (Table S2), as well as several hundred other genomes for which ribotype information was manually retrieved from published data. The correlation between HC150 clustering and ribotyping was high (adjusted Rand coefficient, 0.92; 95% confidence interval, 0.90–0.93). However, our analysis also revealed that PCR ribotypes did not always correspond to phylogenetically coherent groupings. PCR ribotypes 002, 015 and 018 were each distributed across multiple phylogenetic branches (Fig. 8). Furthermore, some genomes with indistinguishable cgMLST alleles were assigned to multiple ribotypes, including RT001/RT241, RT106/RT500 and RT126/RT078 (Fig. 8, Table 1). In these cases, both ribotypes occurred in several, closely related clades (Fig. 8), indicating that similar ribotype banding patterns had evolved multiple times. In contrast, HC150 clusters corresponded to clear-cut phylogenetic groupings within a phylogenetic tree of core genes (Fig. 8b).

### Higher population levels

HierCC can also identify clusters at still higher taxonomic levels, up to the levels of species and sub-species [22]. In *C. difficile *, HC950 clusters seem to correspond to deep evolutionary branches (Fig. S4) and HC2000 clusters were congruent with the major clades reported previously [37], except that cluster HC2000_2 encompassed clade 1 plus clade 2 (Fig. S5). Finally, HC2500 may correspond to the subspecies level, because it distinguished between *C. difficile* and distantly related ‘cryptic clades’ (Fig. S6).

## Discussion

Infectious disease epidemiologists frequently seek to know if new isolates of bacterial pathogens are closely related to others from different geographical origin, i.e. if they are part of a widespread outbreak. Unlike a previous cgMLST implementation [18], EnteroBase supports this goal by taking full advantage of rapidly growing, public repositories of short-read genome sequences [22]. In contrast to short-read archives, however, where stored sequence data are not readily interpretable without specialized bioinformatic tools [38], EnteroBase enables contextual interpretation of a growing collection (18 254 entries as of June 2020) of assembled, quality-controlled *C. difficile* genome sequences and their associated metadata. At least the collection date (year), the geographic origin (country) and the source (host species) are available for the majority of database entries. Importantly, phylogenetic trees based on cgMLST allelic profiles from many thousand bacterial genomes can be reconstructed within a few minutes, whereas such calculations are currently prohibitively slow based on SNP alignments [22]. Genome-sequencing reads from newly sampled *C. difficile* isolates can be uploaded to EnteroBase and compared to all publicly available genome data within hours, without requiring any command-line skills.

We demonstrate that the application of cgMLST to investigations of local *C. difficile* epidemiology yields results that are quantitatively equivalent to those from SNP analyses. This is a major advance because SNP analyses require specific bioinformatic skills and infrastructure, are time consuming and not easily standardized [16]. A web platform for centralized, automated SNP analyses on bacterial genomes is limited to food pathogens currently, and does not offer any analyses on *C. difficile* genomes [39]. Even though a cgMLST scheme for *C. difficile* had been published recently [18], its ability to identify closely related isolates and the inferred genomic distances was shown to be inferior to SNP analyses due to an excess of errors introduced by the *de novo* assembly of sequencing reads and a lack of per-base quality control [20]. In EnteroBase, cgMLST is also based on *de novo* assembly, but EnteroBase uses Pilon [40] to polish the assembled scaffolds and evaluate the reliability of consensus bases of the scaffolds, thereby achieving comparable accuracy to mapping-based SNP analyses. When applied to a large dataset of *C. difficile* genomes from hospital patients in the Oxfordshire region (UK), cgMLST and SNP analysis were largely consistent (89% match) at discriminating between isolates that were sufficiently closely related to have arisen during transmissions chains from others that were epidemiologically unrelated.

After assembly, draft genomes contain missing data and many cgSTs have unique cgST numbers but are identical to other cgSTs, except for missing data. Hence, individual cgST numbers are only rarely informative. However, indistinguishable cgSTs are clustered in common HierCC HC0 clusters, which ignore missing data. In June 2020, the *Clostridioides* database contained >12 000 HC0 clusters, indicating that the majority of genomes was unique. Similarly, EnteroBase provides cluster designations at multiple levels of HierCC, enabling rapid identification of all cgSTs that are related at multiple levels of genetic distance. The data presented here shows that HierCC designations can facilitate communications between researchers, clinicians and public-health officials about transmission chains, epidemic outbreaks, endemic populations and higher phylogenetic lineages up to the level of subspecies.

EnteroBase cgMLST identified numerous HC2 clusters of strains in *C. difficile* isolates that seem to have arisen during transmission chains in six neighbouring hospitals in Germany. These assignments were in part consistent with retrospective investigation of patient location data, although none of the nosocomial outbreaks (defined by German law as two or more infections with likely epidemiological connections [http://www.gesetze-im-internet.de/ifsg/]) had been detected previously by standard epidemiological surveillance by skilled clinical microbiologists. Recent publications propose that prospective genome sequencing of nosocomial pathogens should be applied routinely at the hospital level to guide epidemiological surveillance [41]. Our data indicates that the combination of genome sequencing with cgMLST and HierCC may identify nosocomial transmission routes of *C. difficile* more effectively than presently common practice, and hence could help to reduce pathogen spread and the burden of disease. Reliable identification of transmission chains requires interpretation of pathogen genome sequence data in its epidemiological context, however [42].

HierCC will also enable comparisons to previously published data because we have provided a correspondence table between HC150 clusters and PCR ribotypes (Table 1). Rarefaction analysis indicated that the currently available genome sequences represent about two-thirds of extant HC150 (CC) diversity, which extrapolated to about 600 CCs (Fig. S7). At least some of this enormous diversity may be due to the occupation of multiple, distinct ecological niches, as exemplified by differential propensities for colonizing non-human host species (Table 1) [43, 44]. Individual CCs may also differ in their aptitudes for epidemic spread, as indicated by drastically different proportions of genomes assigned to HC2 chains: only 7% of CC141 were assigned to HC2 clusters versus 35% of CC34 and 77% of CC4 (Table 1). A full understanding of the population structure of *C. difficile* and its relationship to epidemiological patterns will require additional study because many of the clusters described here have not yet been studied or described. However, this task can be addressed by the global community due to the free public access to such an unprecedented amount of genomic data from this important pathogen.

## Methods

### Sampling

In total, 309 *C. difficile* isolates were collected at a diagnostic laboratory providing clinical microbiology services to several hospitals in central Germany. To assemble a representative sample, we included the first 20 isolates from each of six hospitals from each of three consecutive calendar years (Table S2). For investigation of recurrent CDI, a set of 176 *C. difficile* isolates were collected in a diagnostic laboratory in Saarland, Germany. Here, primary stool culture agar plates were stored at 4 °C for 5 months to eventually enable the analysis of multiple plates representing episodes of recurrent *C. difficile* infection from individual patients, who had developed recurrent disease by then and could be chosen with hindsight. It was attempted to pick and cultivate as many bacterial colonies from each selected plate as possible, resulting in 6 to 36 isolates per CDI episode (Table S1). In addition, we sequenced the genomes from 383 isolates that had been characterized by PCR ribotyping previously, including 184 isolates sampled from piglets [8], 71 isolates from various hospitals in Germany [3], and 108 isolates from stool samples collected from nursery home residents (unpublished; Table S2).

### PCR ribotyping

PCR ribotyping was performed as described previously [45], applying an ABI Prism 3100 apparatus for capillary electrophoresis and comparing banding patterns to the Webribo database (https://webribo.ages.at/).

### Whole-genome sequencing

For Illumina sequencing, genomic DNA was extracted from bacterial isolates by using the DNeasy Blood and Tissue kit (Qiagen), and libraries were prepared as described previously [46] and sequenced on an Illumina NextSeq 500 machine using a Mid-Output kit (Illumina) with 300 cycles. For generating complete genome sequences, we applied SMRT long-read sequencing on an RSII instrument (Pacific Biosciences) in combination with Illumina sequencing as reported previously [46]. All genome sequencing data were submitted to the European Nucleotide Archive (www.ebi.ac.uk/ena) under study numbers PRJEB33768, PRJEB33779 and PRJEB33780.

### SNP detection and phylogenetic analysis

Sequencing reads were mapped to the reference genome sequence from *C. difficile* strain R20291 (sequence accession number FN545816) by using BWA-MEM and sequence variation was detected by applying VarScan2 as reported previously [46]. Sequence variation likely generated by recombination was detected through analysis with ClonalFrameML [47] and removed prior to determination of pairwise sequence distances [15] and to construction of maximum-likelihood phylogenetic trees with RAxML (version 8.2.9) [48].

### Genome assembly, quality control and wgMLST allele calling

Genomic data was processed by automated pipelines within EnteroBase, which were described in detail previously [22]. Briefly, Illumina sequencing reads were assembled by using Spades v3.10 [49] and assemblies were improved by applying Pilon [40]. To pass quality control, assemblies were required to comply with the following thresholds: total length, 3.6 to 4.8 Mbp; N50, ≥20 000; number of contigs, ≤600; number of unresolved nucleotides, ≤3%; proportion of *Clostridioides* sequences, >65 % (as determined by Kraken with MiniKraken database [50]). Assemblies were aligned to exemplar alleles by using blastn [51] and the usearch module UblastP [52], and allele numbers, STs and HC numbers assigned by using the EnteroBase module MLSType [22]. All EnteroBase tools are available at https://github.com/zheminzhou/EToKi.

### Statistical analyses

To determine the probability that two genomes are related at ≤2 SNPs, given a certain difference in their cgMLST allelic profiles, we inferred a logistic regression model using R ([53], pp. 593–609). Genomic relatedness was encoded as a binary response variable (1 if ≤2 SNPs, 0 if otherwise) and the number of core-genome allelic differences was used as a predictor variable. We applied this model to a dataset of 1158 genome sequences from a previous study, representing almost all symptomatic CDI patients in Oxfordshire, UK, from 2007 through 2011 [13]. While that original study had encompassed a slightly larger number of sequences, we restricted our analysis to the data (95 %) that had passed quality control as implemented in EnteroBase [21]. We used the SNP data from Eyre's report [13].

The hierarchical single-linkage clustering of cgMLST sequence types was carried out as described [22] for all levels of allelic distances between 0 and 2556. We searched for stable levels of differentiation by HierCC according to the Silhouette index [54], a measure of uniformity of the divergence within clusters. The Silhouette index was calculated based on *d*^', a normalized genetic distance between pairs of STs, which was calculated from their allelic distance *d* as follows: *d*^'=1-(1-*d*)^(1/*l*), where *l* is the average length (937 bp) of the genes in the cgMLST scheme.

We further evaluated the ‘stability’ of hierarchical clustering using two other criteria. The Shannon index is a measure of diversity in a given population. The Shannon index drops from nearly 1 in HC0, because most cgSTs are assigned to a unique HC0 cluster, to 0 in HC2500, which assigns all sequence types to one cluster. The gradient of the Shannon index between the two extremes reflects the frequencies of coalescence of multiple clusters at a lower HC level. Thus, the plateaus in the curve correspond to stable hierarchical levels, where the Shannon index does not change dramatically with HC level. We also evaluated the stability of hierarchical clustering by pairwise comparison of the results from different levels based on the normalized mutual information score [55] (Fig. S3).

For clustering *C. difficile* diversity with PopPUNK [36], we used a sketch size of 10^5^ and a *K* value (maximum number of mixture components) of 15. Of note, the resulting number of clusters for the tested dataset was identical for all *K* between 15 and 30.

To estimate concordance between cgMLST-based hierarchical clustering and PCR ribotyping or PopPUNK clustering, respectively, we calculated the adjusted Rand coefficient [56] by using the online tool available at http://www.comparingpartitions.info/. To test statistical associations of HC2 clusters with specific hospitals and hospital wards, respectively, we compared Χ^2^ values and normalized Shannon entropy values (R package ‘entropy’ v.1.2.1) from contingency tables containing real isolate distributions (Table S3) and randomly permuted distributions (*n*=1000), by using the non-parametric, two-sided Mann–Whitney U test (R package ‘stats’ v.3.5.0).

## Supplementary Data

Supplementary material 1

Supplementary material 2
