# Supplementary material for: A publicly accessible database for Clostridioides difficile genome sequences supports tracing of transmission chains and epidemics
Source: Microb Genom. 2020 Jul 29;6(8):mgen000410. doi: 10.1099/mgen.0.000410 (PMC7641423; doi:10.1099/mgen.0.000410)
Supplement: Supplementary material 1 [file mgen-6-410-s001.pdf]

Supplementary figures.

A publicly accessible database for *Clostridioides difficile* genome sequences supports tracing of transmission chains and epidemics

Martinique Frentrup[1,*], Zhemin Zhou[2,*], Matthias Steglich[1,3], Jan P. Meier-Kolthoff[1], Markus Göker[1], Thomas Riedel[1,3], Boyke Bunk[1], Cathrin Spröer[1], Jörg Overmann[1,3,4], Marion Blaschitz[5], Alexander Indra[5], Lutz von Müller[6], Thomas A. Kohl[7,8], Stefan Niemann[7,8], Christian Seyboldt[9], Frank Klawonn[10,11], Nitin Kumar[12], Trevor D. Lawley[12], Sergio García-Fernández[13,14], Rafael Cantón[13,14], Rosa del Campo[13,14], Ortrud Zimmermann[15], Uwe Groß[15], Mark Achtman[2,§], Ulrich Nübel[1,3,4,§,#]

[*]equal contribution, [§]equal contribution
[#]Corresponding author, email: Ulrich.Nuebel@dsmz.de

[1] Leibniz Institute DSMZ, Braunschweig, Germany
[2] Warwick Medical School, University of Warwick, United Kingdom
[3] German Center for Infection Research (DZIF), Partner site Hannover-Braunschweig, Germany
[4] Braunschweig Integrated Center of Systems Biology (BRICS), Technical University, Braunschweig, Germany
[5] AGES-Austrian Agency for Health and Food Safety, Vienna, Austria
[6] Christophorus-Kliniken, Coesfeld, Germany
[7] Research Center Borstel, Germany
[8] German Center for Infection Research (DZIF), Partner site Hamburg-Lübeck-Borstel, Germany
[9] Friedrich-Loeffler-Institut, Jena, Germany
[10] Biostatistics, Helmholtz Centre for Infection Research, Braunschweig, Germany
[11] Institute for Information Engineering, Ostfalia University, Wolfenbüttel, Germany
[12] Wellcome Sanger Institute, Hinxton, United Kingdom
[13] Servicio de Microbiología, Hospital Universitario Ramón y Cajal, and Instituto Ramón y Cajal de Investigación Sanitaria (IRYCIS), Madrid, Spain
[14] Red Española de Investigación en Patología Infecciosa (REIPI), Madrid, Spain
[15] University Medical Center Göttingen, Germany

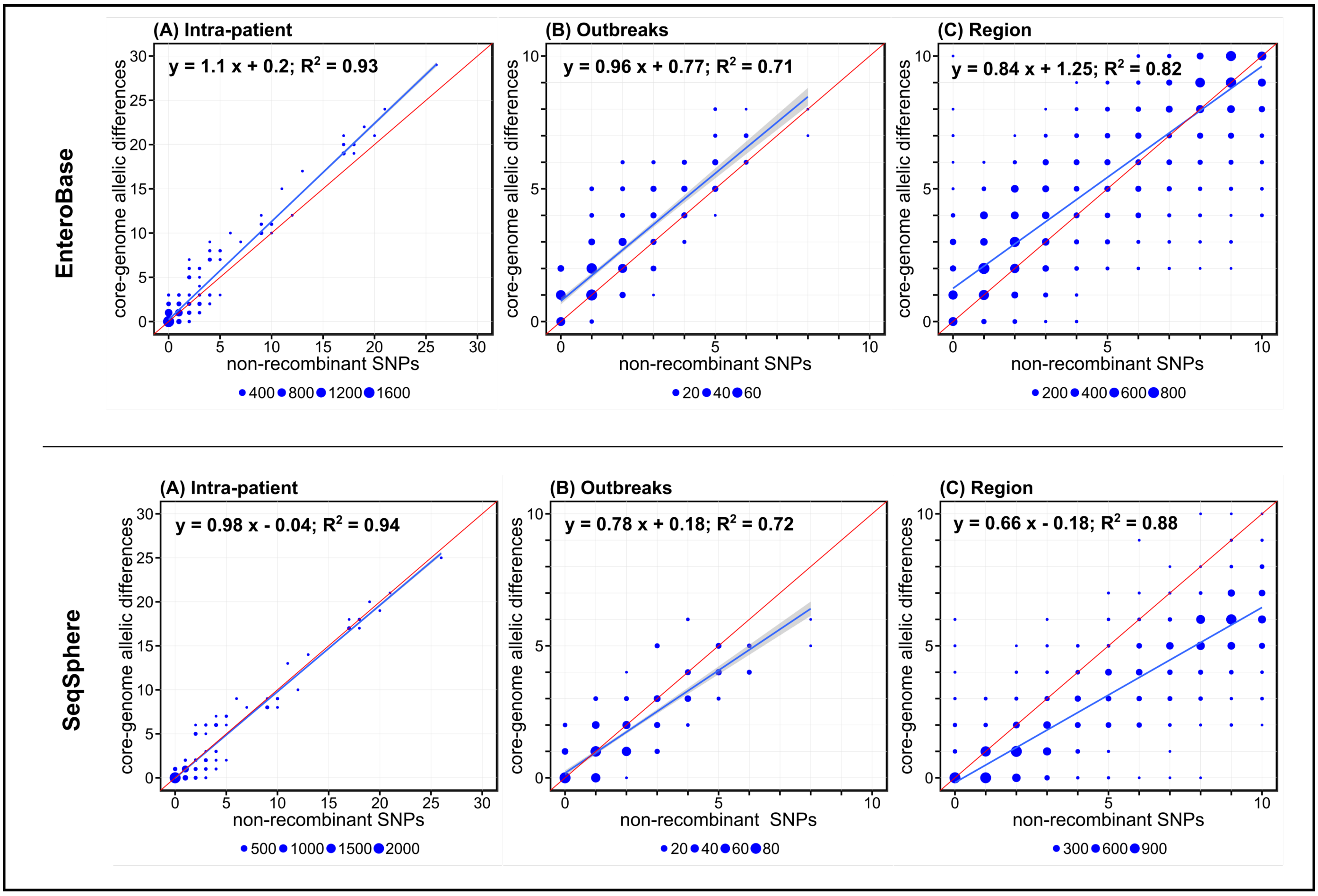

Suppl. Figure 1. Plots of genomic distances from cgMLST versus non-recombinant SNPs. Upper panels are based on EnteroBase cgMLST, lower panels are based on SeqSphere cgMLST [18]. **(A) Intra-patient diversity.** Pairwise genomic distances among 176 isolates from four patients, each of which suffered from two episodes of recurrent CDI, 80 to 153 days apart. **(B) Intra-outbreak diversity.** Pairwise genomic distances among isolates from four previously reported CDI outbreaks, including an outbreak protracting over two years in a hospital in China (number of isolates, n, 12) [19], an outbreak involving two hospitals in Southern Germany (n = 9) [26], and two transmission chains with ribotypes 027 (n=22) and 106/500 (n=20) in a hospital in Madrid, Spain [14]. **(C) Regional diversity.** Pairwise genomic distances among isolates from a comprehensive sample of 1,158 isolates collected from CDI patients in several hospitals in Oxfordshire, UK, between 2007 and 2011 [13]. Distances up to 10 SNPs / 10 cgMLST allelic differences are plotted; the correlation of larger genomic distances is shown in Suppl. Fig. 8.

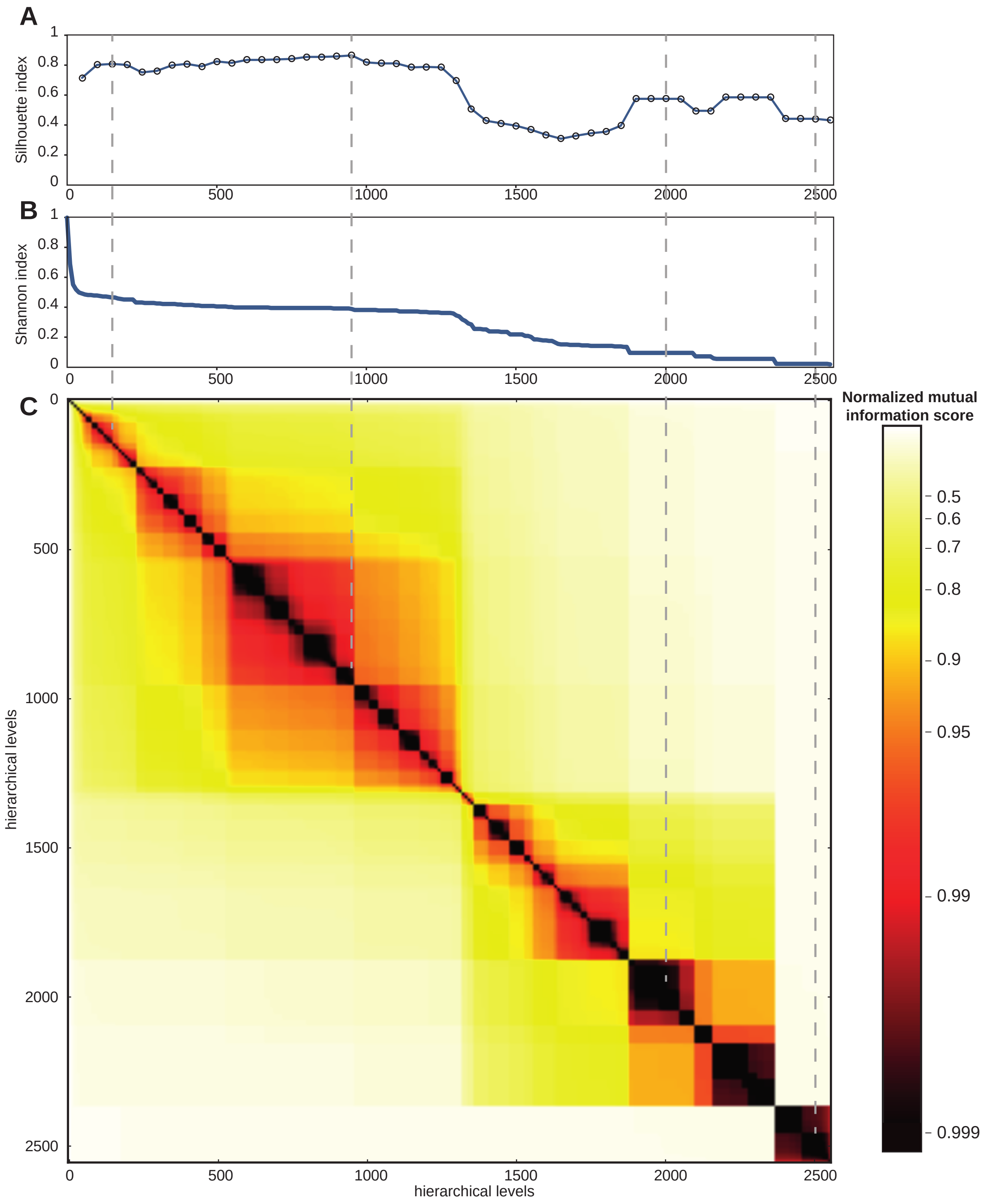

Suppl. Figure 2. Statistical evaluation of hierarchical single-linkage clustering at all levels. Dashed lines indicate hierarchical levels of maximum cluster stability. **(A)** Silhouette index at all hierarchical levels, plotted in steps of 50 allelic differences. Local maxima indicate levels with cohesive, well separated clusters. **(B)** Shannon index at all hierarchical levels. Plateaus in this curve indicate levels with stable clustering. **(C)** Normalized mutual information score comparing the stability of clusters at different hierarchical levels. Dark areas indicate ranges of stable clustering.

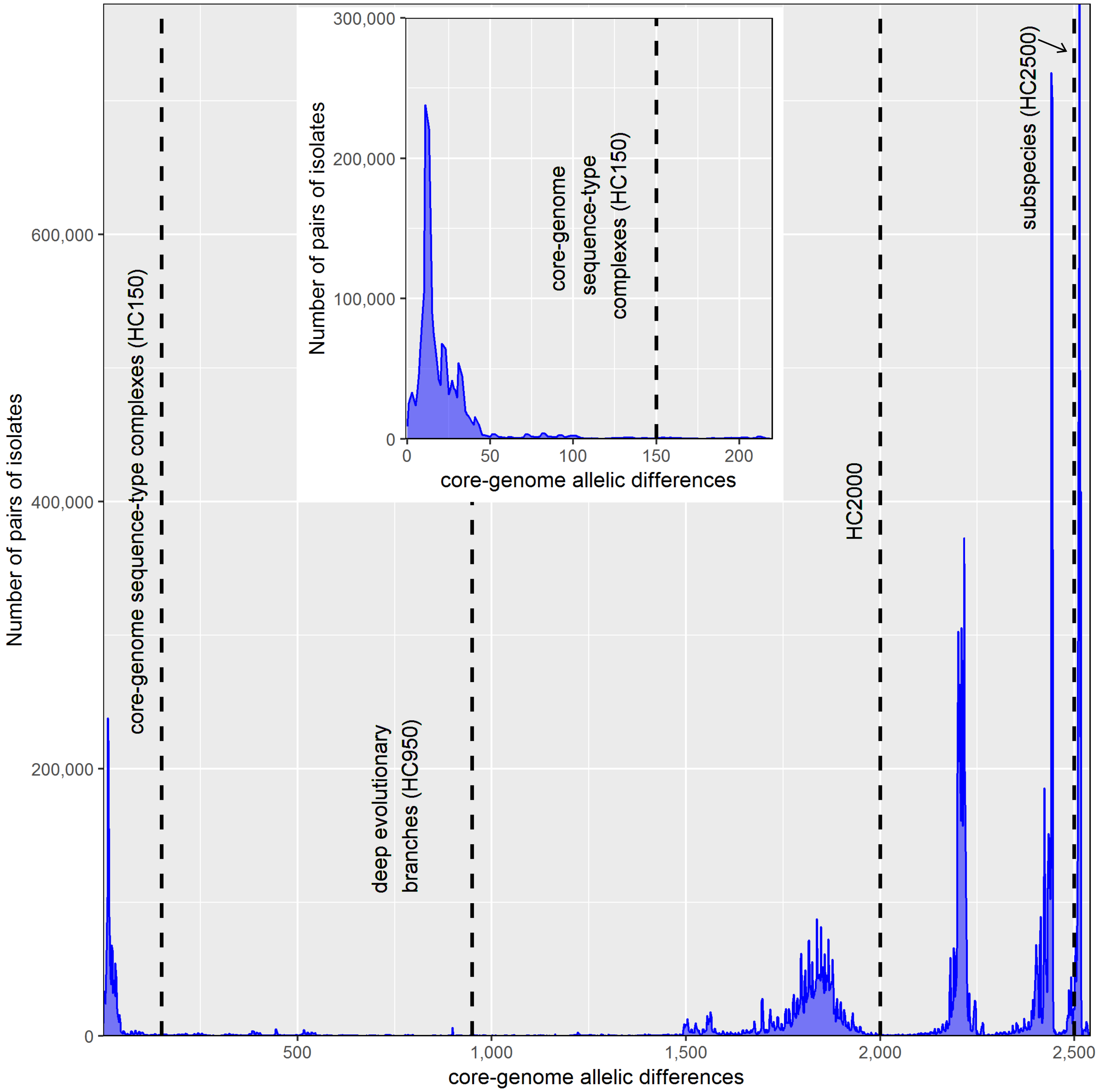

Suppl. Figure 3. Frequency distribution of pairwise genomic distances (cgMLST allelic differences) among 13,515 C. difficile genomes in EnteroBase. Indicated levels of hierarchical clustering are discussed in the text.

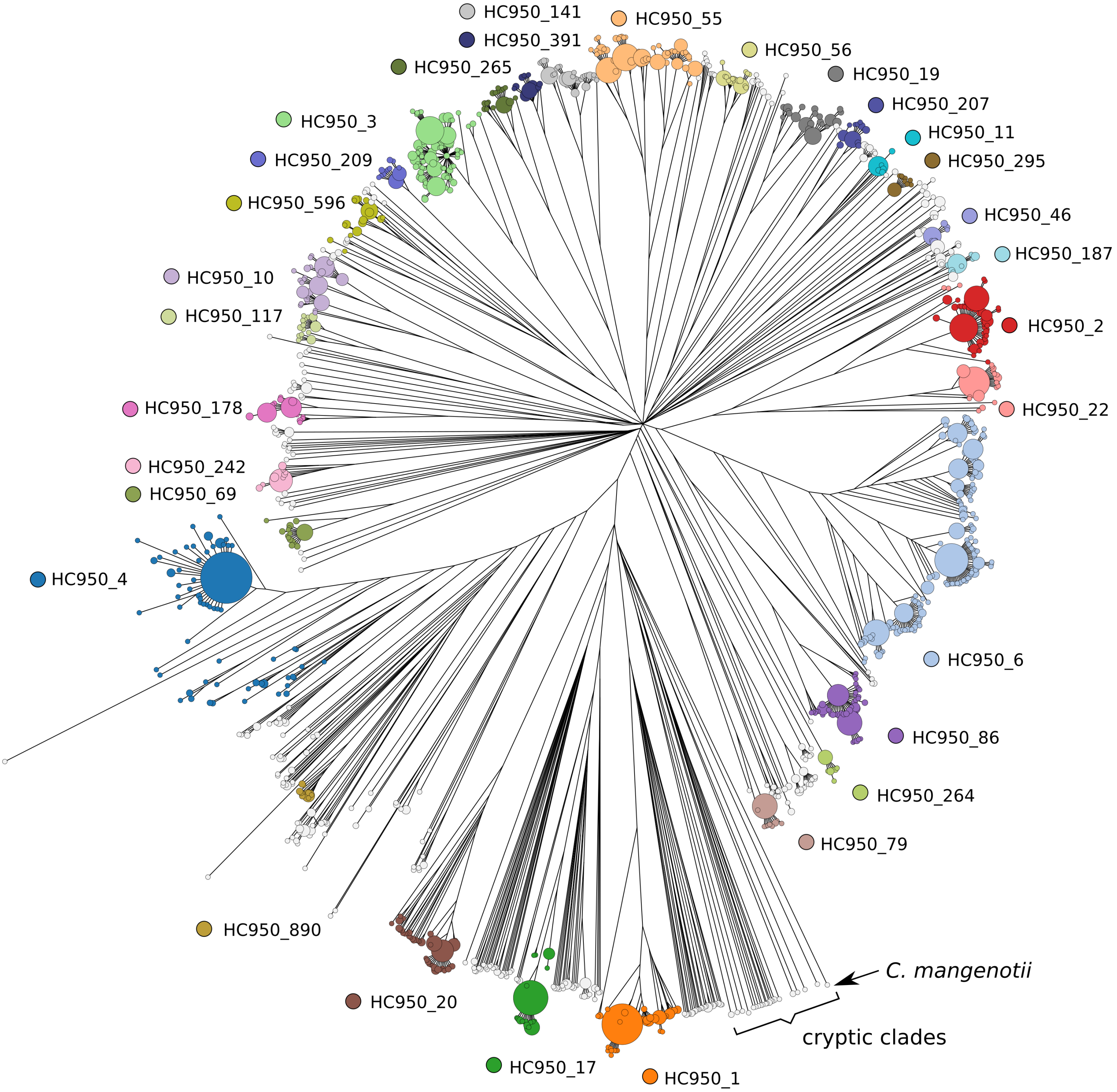

Suppl. Figure 4. Rapid-neighbour-joining phylogenetic tree based on cgMLST variation from 13,515 C. difficile genomes. Colours indicate HC950 clusters.

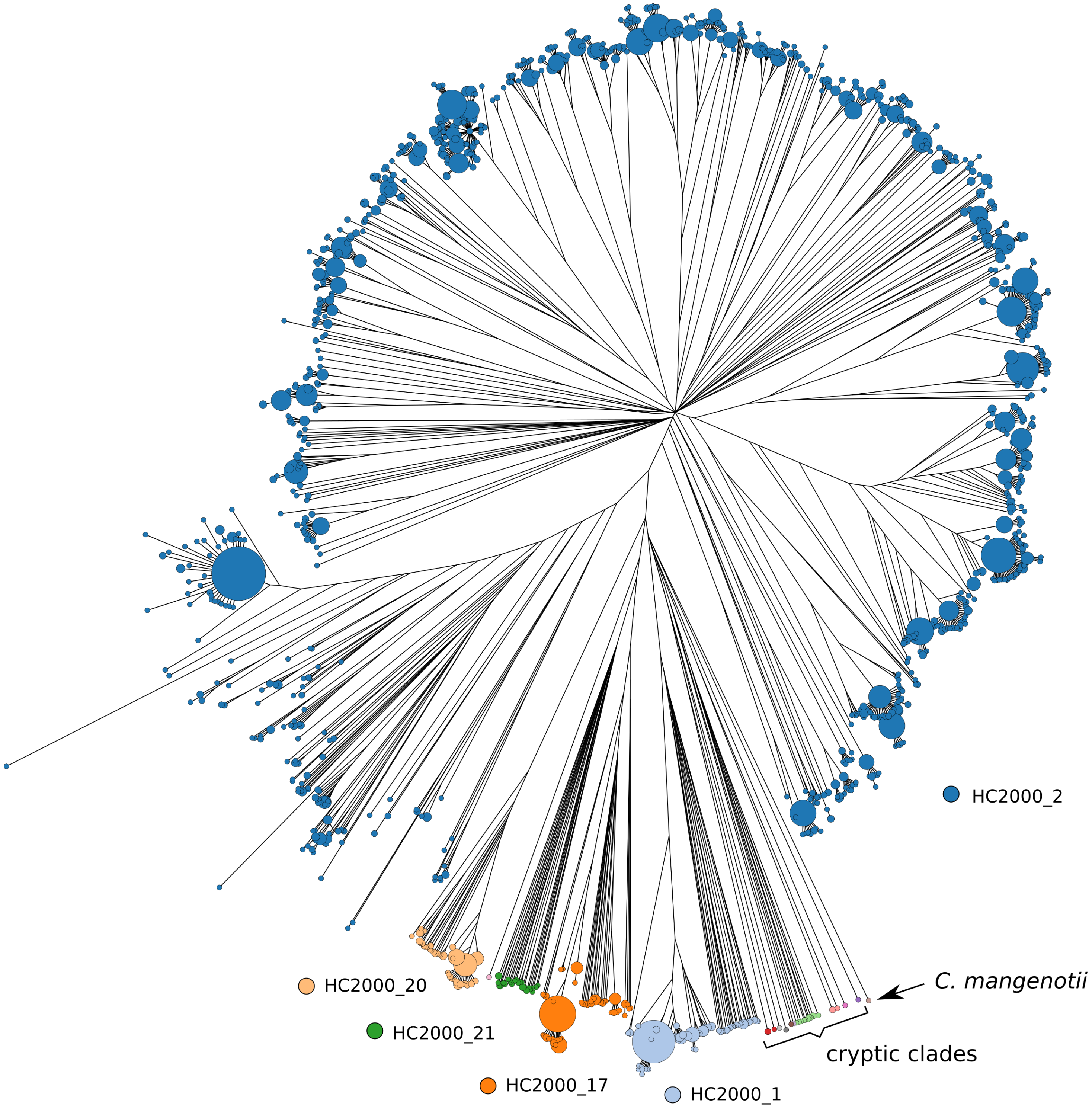

Suppl. Figure 5. Rapid-neighbour-joining phylogenetic tree based on cgMLST variation from 13,515 C. difficile genomes. Colours indicate HC2000 clusters.

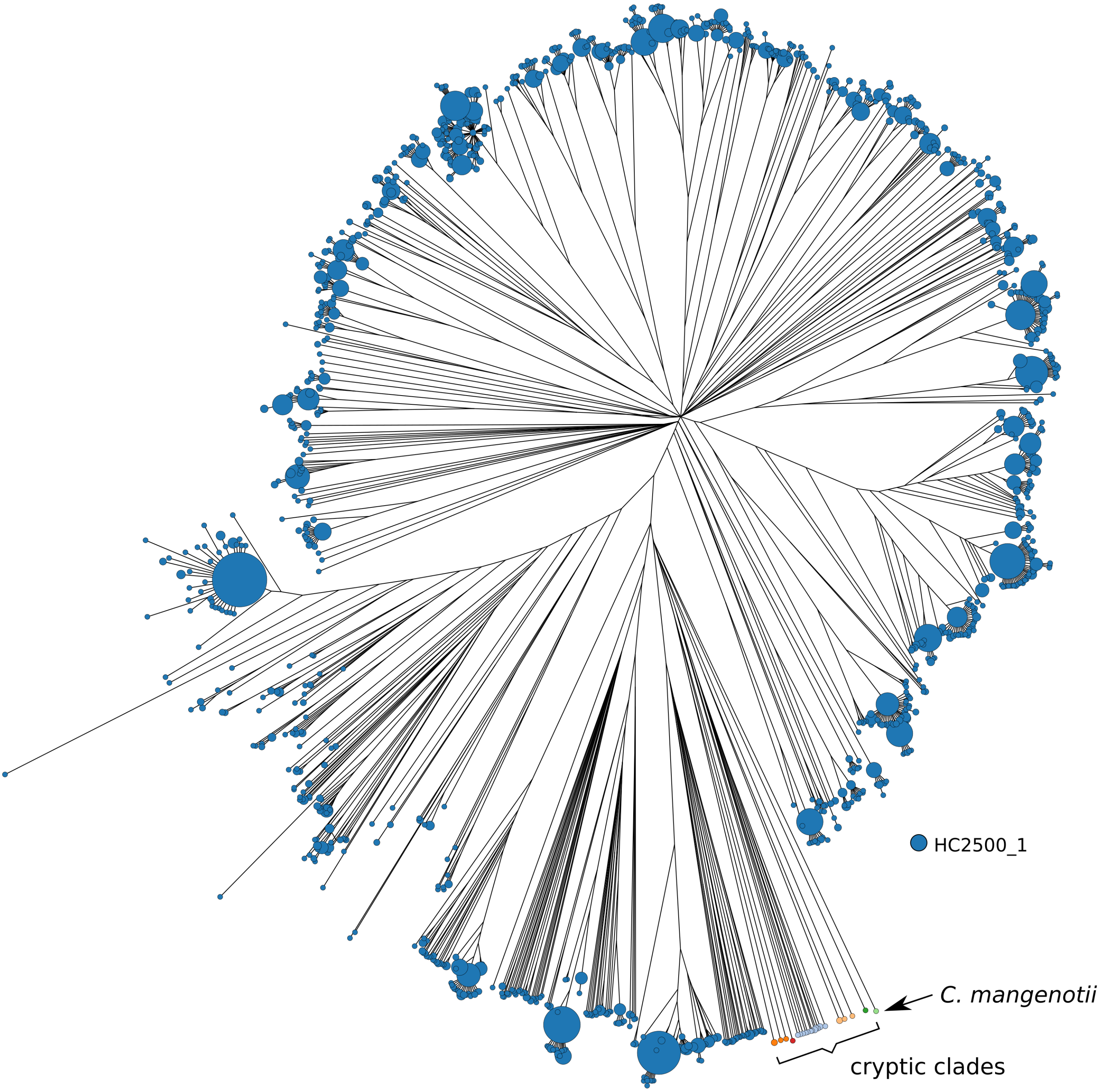

Suppl. Figure 6. Rapid-neighbour-joining phylogenetic tree based on cgMLST variation from 13,515 C. difficile genomes. Colours indicate HC2500 clusters.

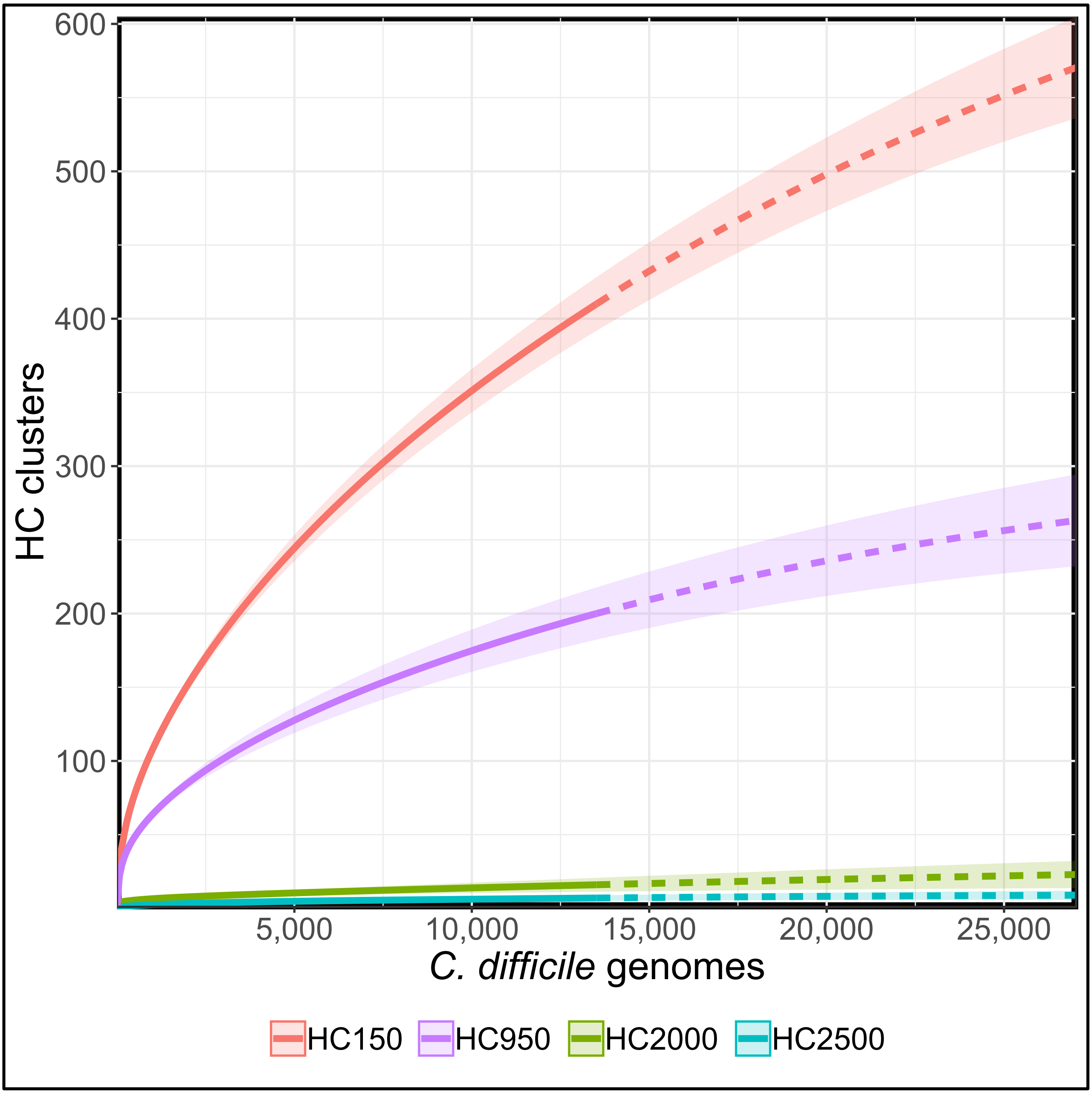

Suppl. Figure 7. Rarefaction analysis to estimate numbers of extant HC clusters.

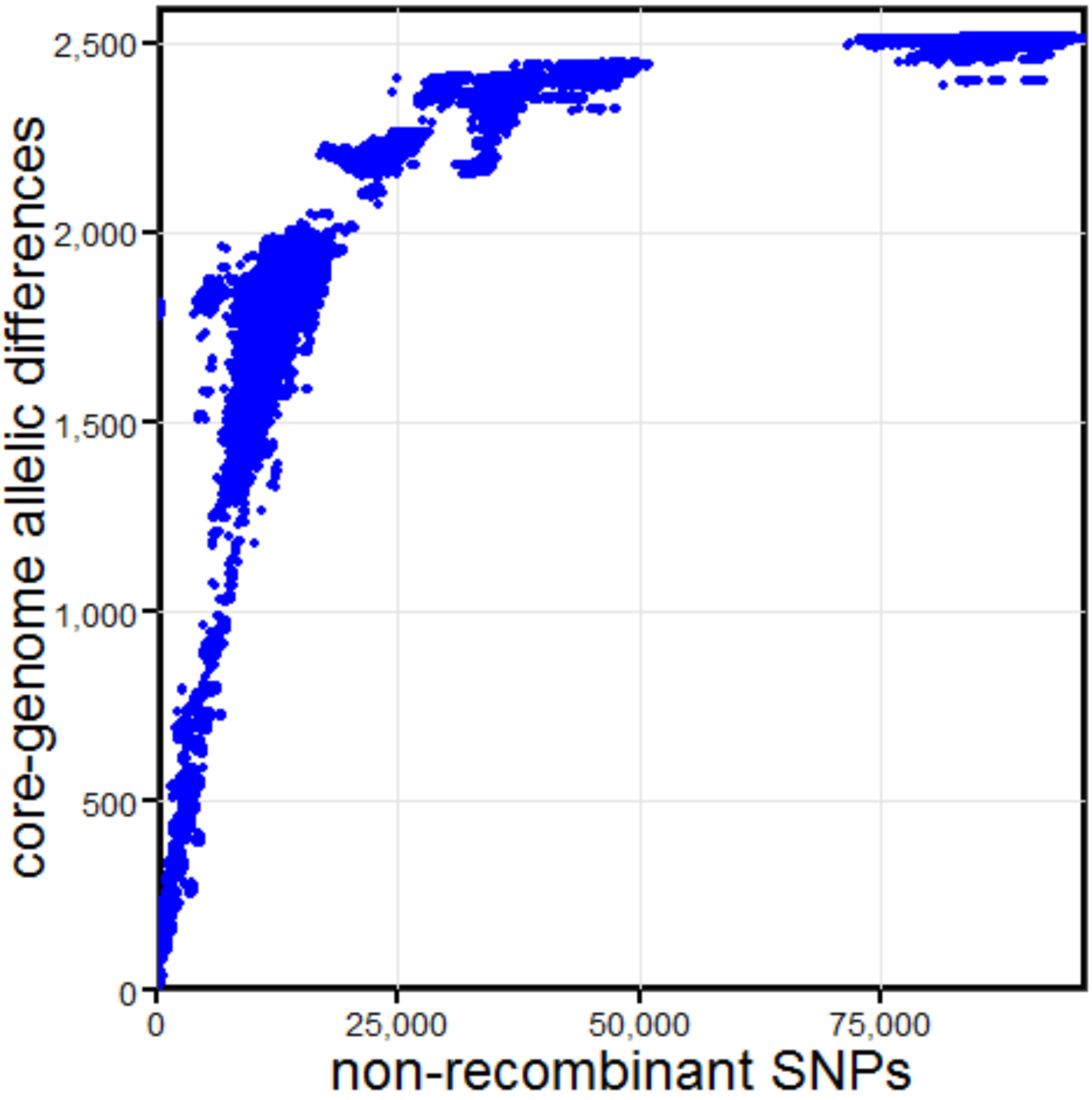

Suppl. Figure 8. Pairwise genomic distances (cgMLST allelic differences versus non-recombinant SNPs) between 1,158 isolates collected from CDI patients in hospitals in Oxfordshire, UK [13].

References.

13. **Eyre DW, Cule ML, Wilson DJ, Griffiths D, Vaughan A, O'Connor L, Ip CL, Golubchik T, Batty EM, Finney JM, Wyllie DH, Didelot X, Piazza P, Bowden R, Dingle KE, Harding RM, Crook DW, Wilcox MH, Peto TE, Walker AS**. Diverse sources of *Clostridium difficile* infection identified on whole-genome sequencing. *N Engl J Med* 2013;369(13):1195-1205.

14. **García-Fernández S, Frentrup M, Steglich M, Gonzaga A, Cobo M, López-Fresneña M, Cobo J, Morosini M-I, Cantón R, del Campo R, Nübel U**. Whole-genome sequencing reveals nosocomial *Clostridioides difficile* transmission and a previously unsuspected epidemic scenario. *Sci Rep* 2019;9:6959.

19. **Jia H, Du P, Yang H, Zhang Y, Wang J, Zhang W, Han G, Han N, Yao Z, Wang H, Zhang J, Wang Z, Ding Q, Qiang Y, Barbut F, Gao GF, Cao Y, Cheng Y, Chen C**. Nosocomial transmission of *Clostridium difficile* ribotype 027 in a Chinese hospital, 2012-2014, traced by whole genome sequencing. *BMC Genomics* 2016;17:405.

26. **Berger FK, Gfrörer S, Becker SL, Baldan R, Cirillo DM, Frentrup M, Steglich M, Engling P, Nübel U, Mellmann A, Bischoff M, Gartner B, von Müller L**. Hospital outbreak due to *Clostridium difficile* ribotype 018 (RT018) in Southern Germany. *Int J Med Microbiol* 2019;309:189-193.
